# Changes in Smoking Cessation–Related Behaviors Among US Adults During the COVID-19 Pandemic

**DOI:** 10.1001/jamanetworkopen.2022.25149

**Published:** 2022-08-01

**Authors:** Priti Bandi, Samuel Asare, Anuja Majmundar, Zheng Xue, Xuesong Han, J. Lee Westmaas, Nigar Nargis, Ahmedin Jemal

**Affiliations:** 1 Surveillance and Health Equity Science, American Cancer Society, Atlanta, Georgia; 2 Population Science, American Cancer Society, Atlanta, Georgia

## Abstract

**Importance:**

Smoking cessation is an urgent public health priority given that smoking is associated with increased risk of severe COVID-19 outcomes and other diseases. It is unknown how smoking cessation changed nationally during the COVID-19 pandemic.

**Objective:**

To investigate changes in smoking cessation–related behaviors in the US during the COVID-19 pandemic.

**Design, Setting, and Participants:**

This cross-sectional study was conducted using 2011 to 2020 data on 788 008 individuals who had smoked in the past year from the nationally representative Behavioral Risk Factor Surveillance System (BRFSS) survey. Representative retail scanner sales data between January 2017 and July 2021 for 1004 unique nicotine replacement therapy (NRT) universal product codes in 31 US states from NielsenIQ were also used.

**Exposures:**

Calendar year and 4-week sales periods.

**Main Outcomes and Measures:**

Changes in annual self-reported prevalence of past-year quit attempts and recent successful cessation before (ie, 2011-2019) and during (ie, 2020) the COVID-19 pandemic and changes in sales volumes in millions of pieces of nicotine gum, lozenge, and patch brands before (1271 four-week sales periods between January 2017 and February 2020) and during (558 four-week sales periods between March 2020 and July 2021) the COVID-19 pandemic were calculated.

**Results:**

The 2011 to 2020 pooled BRFSS sample (response rate range, 45.2%-49.9%) included 788 008 respondents (243 061 individuals ages 25-44 years [weighted percentage, 42.5%]; 374 519 men [weighted percentage, 55.7%]). For the first time since 2011, annual past-year quit attempt prevalence decreased between 2019 and 2020, from 65.2% (95% CI, 64.5% to 65.9%) to 63.2% (95% CI, 62.3% to 64.0%), with the largest relative decreases among individuals ages 45 to 64 years (61.4% [95% CI, 60.3% to 62.5%] vs 57.7% [95% CI, 56.3% to 59.2%]), those with 2 or more comorbidities (67.1% [95% CI, 66.0% to 68.2%] to 63.0% [95% CI, 61.6% to 64.4%]), and Black individuals (72.5% [95% CI, 70.3 to 74.6] vs 68.4% [95% CI, 65.3% to 71.3%]). Recent successful cessation remained unchanged during 2019 to 2020. Observed mean (SD) 4-week NRT sales volume in the prepandemic period was 105.6 (66.2) million gum pieces, 51.9 (31.6) million lozenges, and 2.0 (1.1) million patches. Compared with expected sales, observed sales during the COVID-19 pandemic were lower by 13.0% (95% CI, –13.7% to –12.3%) for lozenges, 6.4% (95% CI, –7.3% to –5.5%) for patches, and 1.2% (95% CI, –1.7% to –0.7%) for gum.

**Conclusions and Relevance:**

This study found that serious smoking cessation activity among US adults decreased immediately and remained depressed for more than a year during the COVID-19 pandemic. These findings suggest that smokers experiencing disproportionately negative outcomes during the pandemic should be reengaged and assisted in quit attempts.

## Key Points

**Question:**

Did smoking cessation–related behaviors change during the COVID-19 pandemic in the US?

**Findings:**

This cross-sectional study among 788 008 US adult smokers found that the annual prevalence of past-year quit attempts decreased for the first time since 2011, from 65.2% in 2019 to 63.2% in 2020. Simultaneously, observed sales of nicotine replacement therapy brands from representative retail scanner data across 31 US states decreased by 1% to 13% compared with expected sales.

**Meaning:**

These findings suggest a decrease in smoking cessation activity during the COVID-19 pandemic and the need to reengage smokers in evidence-based quitting strategies.

## Teaser

This cross-sectional study compares prevalence of past-year quit attempts and sales volumes of nicotine replacement therapy before vs during the COVID-19 pandemic among US adult smokers.

## Introduction

Smoking cessation is associated with reduced risk of 12 cancers and adverse cardiovascular, reproductive, and respiratory conditions caused by smoking.^1^ Promoting cessation is a current public health priority given that smoking is associated with increased risk of severe COVID-19 outcomes.^2^ However, it is unknown how smoking cessation changed nationally during the COVID-19 pandemic. Existing evidence has been mixed and generally based on nonrepresentative samples. Studies suggest that stress-related coping increases in cigarette smoking occurred among some smokers, whereas fears of COVID-19 health risks may have been associated with some individuals’ decisions to reduce use of or quit tobacco products.^3,4,5,6,7^ A 2022 analysis^8^ of nationally representative cross-sectional data from the Behavioral Risk Factor Surveillance System (BRFSS) survey reported a decrease in smoking prevalence during the COVID-19 pandemic but did not assess changes in smoking cessation behaviors. To address these gaps, we used 2 distinct but complementary data sources to investigate changes in past-year quit attempts, recent successful cessation, and sales of nicotine replacement therapies (NRTs; effective cessation treatments used by 25% of US smokers in quit attempts^1,9^) before and during the COVID-19 pandemic in the US.

## Methods

This cross-sectional study followed the Strengthening the Reporting of Observational Studies in Epidemiology (STROBE) reporting guideline. This study was based on data that were deidentified, publicly available, or not from human participants, which the US Department of Health and Human Services considers as nonhuman research that does not require institutional review board review or informed consent.

### Data

Cessation behaviors from the BRFSS, an annual, nationally representative, cross-sectional, telephone-based health survey of adults (those ages ≥18 years) who were not institutionalized, were examined for 2011 to 2020. The response rate ranged from 45.2% to 49.9% and was 49.4% in 2019 and 47.9% in 2020.^10^ The analytic sample in the pooled data from 2011 to 2020 included individuals aged 18 years or older who currently smoked every day or some days and former smokers who had quit within the past year with complete data on question assessing past-year quit attempts (data missing for 179 257 of 4 563 068 respondents [3.9%]).

NielsenIQ Retail Scanner Data provided representative state-level sales data from US-based retail channels, including grocery, supermarket, mass merchandise, drug, convenience (chain, franchise, and independent), dollar, and club stores in 33 US states. After 2 states with missing NRT sales data were excluded, the analytic sample included 1004 unique universal product codes (UPCs) of NRT brands for 59 four-week sales periods, from January 2017 to July 2021, for each of 31 US states.

### Outcomes

Population-level cessation behaviors were self-reported past-year quit attempts (ie, current smokers who reported that they stopped smoking for >1 day during the past 12 months because they were trying to quit smoking or former smokers who quit during the past year; missing data for 2881 of 788 008 respondents [0.4%]) and recent successful cessation (ie, former smokers who reported last smoking a cigarette, “even 1 or 2 puffs,” >6 months but <1 year before the survey).

The NRT sales outcome was state-level aggregate 4-week sales volumes of nicotine gum, lozenge, and patch brand UPCs in millions of pieces before (1271 four-week sales periods between January 2017 and February 2020) and during (558 four-week sales periods between March 2020 and July 2021) the COVID-19 pandemic. NRT product types were assessed individually given that information on usage patterns (eg, frequency and combination use) were unknown.

### Statistical Analysis

Annual prevalence and 95% CIs of cessation behaviors were estimated overall, by interview quarter (1-4), sociodemographic factors previously reported to be associated with smoking cessation behaviors (age, sex, self-reported race and ethnicity, education level, marital status, and region), and health-associated factors (number of comorbidities; self-rated health; past 30-day frequency of mental distress, physical distress, and activity limitations; smokeless tobacco use; heavy alcohol drinking [self-report of >14 drinks/wk among men and >7 drinks/wk among women in 30 days before survey]; and body mass index category [BMI; calculated as weight in kilograms divided by height in meters squared]). The BRFSS survey provided a combined race and ethnicity variable (American Indian or Alaska Native only, non-Hispanic; Asian only, non-Hispanic; Black, non-Hispanic; Hispanic; White, non-Hispanic) based on exclusive categorizations of self-reported ethnicity (Hispanic, Latino or Latina, or Spanish origin or not) and race categories. Multivariate logistic regression models were used to test for linear (continuous year), quadratic (year squared), and cubic (year cubed) time trends over 2011 to 2020 and to estimate annual relative difference (% change) and absolute difference (percentage point change) of estimated marginal probabilities between 2020 (assumed to be during the COVID-19 pandemic) and past survey years adjusted for interview quarter and sociodemographic and health-associated factors (eTable 1 in the Supplement). Additionally, regression models were stratified by sociodemographic and health-associated variables to assess differences in trends across subgroups during the COVID-19 pandemic. Regression analyses were stratified by interview quarter to assess more granular trends during the COVID-19 pandemic vs past years. Cessation behaviors in 2020 were compared with those before the COVID-19 pandemic onset (ie, 2020 quarter 1) and during the COVID-19 pandemic (ie, 2020 quarters 2-4) vs corresponding periods in 2019. Estimates were weighted to be nationally representative and accounted for sampling strategy.

Interrupted time series regression analysis was used to estimate expected NRT sales volume during the COVID-19 pandemic (March 2020 to July 2021) based on trends estimated from the prepandemic period (January 2017 to February 2020). Changes in NRT sales during the COVID-19 pandemic were calculated as the relative difference between observed and expected sales volumes. Regression analyses controlled for state and month fixed effects, inflation-adjusted NRT and cigarette prices, and state sociodemographic composition (sex, marital status, age, race and ethnicity, education, and household income).^11^

Analyses were conducted using Stata statistical software version 17.0 (StataCorp) and SAS-callable SUDAAN statistical software version 9.4 (SAS Institute). All statistical tests were 2-sided, with significance set at *P* < .05.

## Results

### Changes in Prevalence of Smoking Cessation Behaviors

The 2011 to 2020 pooled sample included 788 008 respondents (243 061 individuals ages 25-44 years [42.5%; 95% CI, 42.3%-42.7%] and 346 915 individuals ages 45-64 years [35.5%; 95% CI, 35.3%-35.7%]; 374 519 [55.7%; 95% CI, 55.5%-55.9%] men). There were 23 763 American Indian or Alaska Native individuals (1.8%; 95% CI, 1.7%-1.8%), 9614 Asian individuals (2.5%; 95% CI, 2.4%-2.6%), 69 638 Black individuals (12.7%; 95% CI, 12.6%-12.9%), 56 829 Hispanic individuals (14.1%; 95% CI, 13.9%-14.3%), and 583 306 White individuals (68.9%; 95% CI, 68.7%-69.2%) (eTable 1 in the Supplement). Between 2019 and 2020, there was a significant increase in percentage of individuals ages 65 years or older (14 445 of 67 425 individuals [12.0%; 95% CI, 11.7%-12.4%] to 12 811 of 62 480 individuals [12.8%; 95% CI, 12.3%-13.3%]; *P* = .04); the percentage of Northeast residents increased (9973 of 66 334 individuals [13.9%; 95% CI, 13.4%-14.3%] to 10 527 of 61 578 individuals [15.4%; 95% CI, 14.9%-15.9%]), while the percentage of Western region residents decreased (13 974 of 66 334 individuals [20.3%; 95% CI, 19.7%-20.9%] to 12 983 of 61 578 individuals [19.1%; 95% CI, 18.3%-19.9%]) (*P* < .001) (eTable 1 in the Supplement). The percentage of individuals interviewed in 2020 quarters 2 and 3 was significantly lower than those in 2019 quarters 2 and 3, whereas more people were interviewed in 2020 quarter 1 and quarter 4 vs the corresponding quarters in 2019 (eTable 1 in the Supplement).

**Figure 1.**
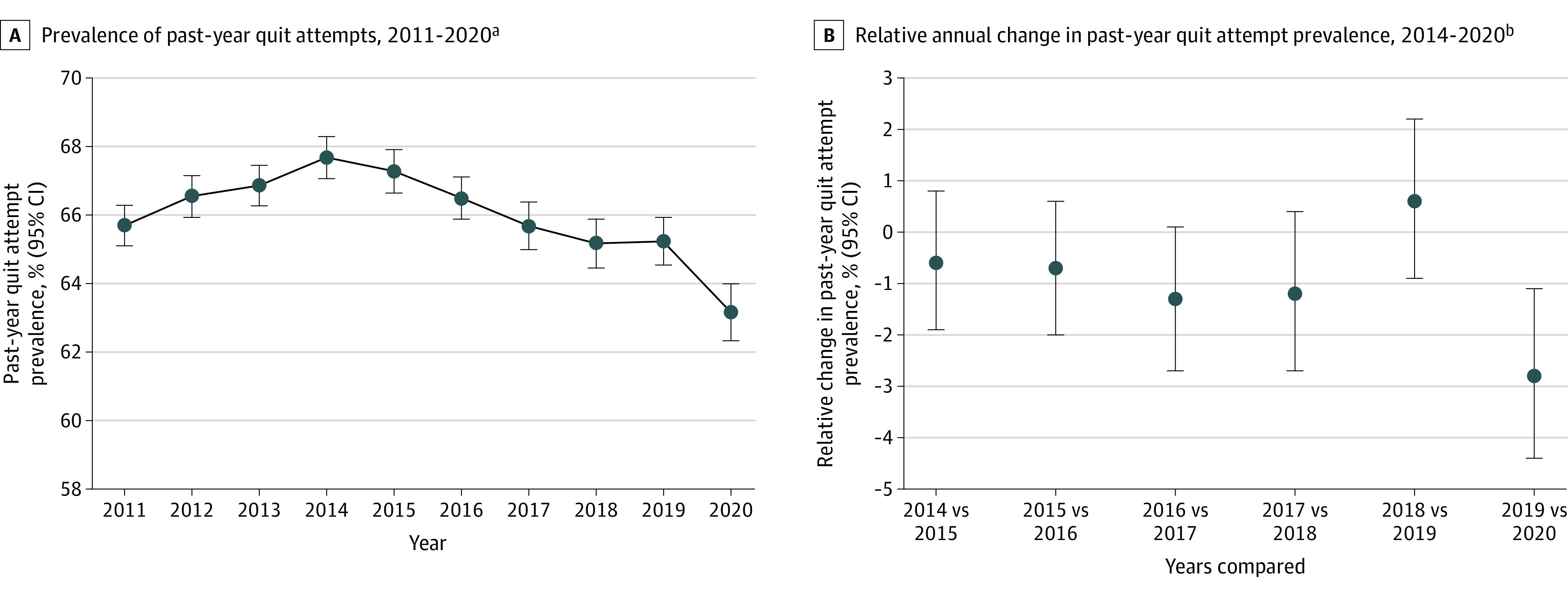
Prevalence of Past-Year Quit Attempts ^a^Estimated as a weighted percentage, where the numerator (consisting of the number of current smokers [ie, those who smoked ≥100 cigarettes in their lifetime and now smoke every day or somedays] who reported that they had stopped smoking for >1 day during the past 12 months because they were trying to quit smoking and former smokers [ie, ever smokers who currently smoked “not at all”] who quit during the past year [ie, last smoked a cigarette, “even 1 or 2 puffs,” within the past year]) was divided by the denominator (consisting of current smokers and former smokers who quit within the past year) and multiplied by 100. ^b^Estimated as the ratio of current year vs past year estimated marginal probabilities minus 1, multiplied by 100, from a logistic regression model estimating the probability of past-year quit attempts between 2011 and 2020, adjusted for age, sex, race and ethnicity, education level, marital status, region, number of comorbidities, smokeless tobacco use, heavy alcohol drinking, past 30-day mental distress frequency, body mass index category (calculated as weight in kilograms divided by height in meters squared), and interview quarter. Zero percent indicates null change between years compared.

Past-year quit attempt prevalence followed a quadratic trend (*P* < .001), peaking at 67.7% (95% CI, 67.1% to 68.3%) in 2014 (Figure 1A). Between 2019 and 2020, prevalence decreased 2.8% (95% CI, –4.5% to –1.0%), from 65.2% (95% CI, 64.5% to 65.9%) to 63.2% (95% CI, 62.3% to 64.0%) (*P* = .002), whereas no past annual change after 2014 was significant (Figure 1B). Quit attempt prevalence was lower in 2020 compared with 2019 for quarter 2 (62.7% [95% CI, 61.0% to 64.3%] vs 66.1% [95% CI, 64.7% to 67.5%]; *P* = .009) and remained low but not significantly different in quarter 3 (63.1% [95% CI, 61.5% to 64.7%] vs 65.3% [95% CI, 63.9% to 66.6%]; *P* = .05) and quarter 4 (63.2% [95% CI, 61.6% to 64.9%] vs 65.2% [95% CI, 63.9% to 66.6%]; *P* = .09) (Table). In order of relative magnitude, the largest statistically significant decreases in quit attempt prevalence between 2019 and 2020 were among individuals ages 45 to 64 years (61.4% [95% CI, 60.3% to 62.5%] to 57.7% [95% CI, 56.3% to 59.2%]; *P* < .001), those with 2 or more comorbidities (67.1% [95% CI, 66.0% to 68.2%] to 63.0% [95% CI, 61.6% to 64.4%]; *P* < .001), Black individuals (72.5% [95% CI, 70.3% to 74.6%] to 68.4% [95% CI, 65.3% to 71.3%]; *P* = .02), those with frequent (≥14 days) past 30-day activity limitations (69.2% [95% CI, 67.6% to 70.7%] vs 65.0% [95% CI, 62.9% to 67.0%]; *P* = .007) and physical distress (65.4% [95% CI, 64.7% to 66.1%] to 63.3% [95% CI, 62.4% to 64.1%]; *P* = .01), individuals with high school degrees or less (63.6% [95% CI, 62.6% to 64.6%] to 60.6% [95% CI, 59.4% to 61.7%]; *P* = .003), Midwestern residents (63.2% [95% CI, 62.0% to 64.4%] to 60.3% [95% CI, 59.1% to 61.6%]; *P* = .02), White individuals (62.3% [95% CI, 61.5% to 63.1%] to 59.8% [95% CI, 58.9% to 60.7%]; *P* = .001), women (65.2% [95% CI, 64.3% to 66.2%] to 63.2% [95% CI, 62.0% to 64.3%]; *P* = .01), individuals rating their general health as excellent or very good (65.7% [95% CI, 64.5% to 66.8%] to 63.6% [95% CI, 62.3% to 64.9%]; *P* = .007), and those reporting infrequent (0 to <14 days) past 30-day mental distress (64.8% [95% CI, 64.0% to 65.6%] to 62.7% [95% CI, 61.7% to 63.6%]; *P* = .02) (Table). Quit attempt prevalence changes between 2019 and 2020 did not vary across subgroups of heavy alcohol use, smokeless tobacco use, or BMI.

**Table.** Prevalence of Past-Year Quit Attempts

Characteristic	Past-year quit attempts, % (95% CI)^a^				2020 vs 2019		
	2017 (n = 75 358)	2018 (n = 73 159)	2019 (n = 67 425)	2020 (n = 62 480)	Relative difference (95% CI), %^b^	Absolute difference (95% CI), percentage points^c^	*P* value
Total	65.7 (65.0 to 66.4)	65.2 (64.4 to 65.9)	65.2 (64.5 to 65.9)	63.2 (62.3 to 64.0)	–2.8 (–4.5 to –1.0)	–1.8 (–3.0 to –0.6)	.002
Quarter							
1	66.3 (64.8 to 67.9)	64.4 (62.8 to 65.9)	63.7 (61.9 to 65.4)	63.6 (61.7 to 65.4)	0.5 (–3.5 to 4.6)	–0.0 (–2.6 to 2.6)	.83
2	65.3 (63.9 to 66.7)	66.0 (64.4 to 67.5)	66.1 (64.7 to 67.5)	62.7 (61.0 to 64.3)	–4.5 (–7.7 to –1.1)	–2.9 (–5.1 to –0.8)	.009
3	65.7 (64.3 to 67.0)	65.7 (64.2 to 67.1)	65.3 (63.9 to 66.6)	63.1 (61.5 to 64.7)	–3.2 (–6.4 to 0.0)	–2.1 (–4.2 to 0.1)	.05
4	65.7 (64.2 to 67.1)	64.5 (63.1 to 65.9)	65.2 (63.9 to 66.6)	63.2 (61.6 to 64.9)	–2.8 (–6.0 to 0.5)	–1.8 (–4.0 to 0.4)	.09
Age-group, y							
18-24	75.1 (72.7 to 77.4)	74.6 (72.2 to 76.9)	74.1 (71.3 to 76.7)	77.1 (74.2 to 79.8)	3.7 (–1.5 to 9.2)	3.0 (–0.9 to 6.9)	.17
25-44	68.2 (67.0 to 69.3)	69.1 (68.0 to 70.2)	68.4 (67.3 to 69.6)	66.8 (65.4 to 68.1)	–1.7 (–4.3 to 0.9)	–1.2 (–3.0 to 0.6)	.20
45-64	62.3 (61.2 to 63.3)	60.2 (58.9 to 61.4)	61.4 (60.3 to 62.5)	57.7 (56.3 to 59.2)	–6.4 (–9.3 to –3.4)	–3.9 (–5.7 to –2.1)	<.001
≥65	58.0 (56.2 to 59.7)	57.1 (55.2 to 58.9)	57.8 (56.1 to 59.6)	56.2 (54.2 to 58.3)	–2.6 (–7.2 to 2.2)	–1.5 (–4.2 to 1.2)	.27
Sex							
Women	66.4 (65.4 to 67.4)	65.3 (64.3 to 66.3)	65.2 (64.3 to 66.2)	63.2 (62.0 to 64.3)	–3.1 (–5.4 to –0.7)	–2.0 (–3.6 to –0.4)	.01
Men	65.1 (64.1 to 66.1)	65.1 (64.1 to 66.0)	65.2 (64.2 to 66.2)	63.2 (62.0 to 64.3)	–2.5 (–4.9 to 0.0)	–1.6 (–3.2 to –0.0)	.05
Race and ethnicity							
American Indian or Alaska Native	67.0 (62.4 to 71.2)	56.3 (50.6 to 61.9)	62.0 (57.7 to 66.2)	60.9 (55.7 to 65.9)	–0.8 (–11.0 to 10.6)	–0.5 (–7.4 to 6.4)	.89
Asian	76.6 (71.2 to 81.2)	76.3 (71.0 to 80.8)	72.2 (66.2 to 77.4)	73.1 (65.7 to 79.4)	0.6 (–10.6 to 13.2)	–0.0 (–8.6 to 8.6)	.92
Black	74.0 (72.0 to 75.8)	72.6 (70.3 to 74.8)	72.5 (70.3 to 74.6)	68.4 (65.3 to 71.3)	–5.7 (–10.3 to –0.8)	–4.1 (–7.6 to –0.6)	.02
Hispanic	71.3 (68.7 to 73.6)	69.7 (66.9 to 72.4)	71.6 (68.9 to 74.1)	72.4 (69.5 to 75.1)	1.3 (–4.1 to 6.9)	1.0 (–2.9 to 4.9)	.65
White	62.5 (61.7 to 63.3)	62.4 (61.6 to 63.2)	62.3 (61.5 to 63.1)	59.8 (58.9 to 60.7)	–3.3 (–5.1 to –1.4)	–2.0 (–3.2 to –0.8)	.001
Education level							
≤High school	62.7 (61.7 to 63.7)	63.5 (62.5 to 64.5)	63.6 (62.6 to 64.6)	60.6 (59.4 to 61.7)	–3.9 (–6.4 to –1.4)	–2.5 (–4.1 to –0.9)	.003
Some college	69.0 (67.8 to 70.2)	66.5 (65.2 to 67.8)	66.3 (65.1 to 67.5)	65.7 (64.3 to 67.2)	–0.5 (–3.4 to 2.4)	–0.4 (–2.4 to 1.6)	.71
College	70.8 (69.3 to 72.2)	69.0 (67.3 to 70.5)	69.6 (68.1 to 71.1)	68.0 (66.1 to 69.8)	–2.2 (–5.5 to 1.2)	–1.6 (–4.0 to 0.8)	.19
Region							
Northeast	66.5 (64.9 to 68.1)	67.5 (65.8 to 69.2)	64.4 (62.7 to 66.1)	64.6 (62.9 to 66.2)	0.0 (–3.7 to 3.8)	–0.0 (–2.4 to 2.4)	.99
Midwest	62.8 (61.6 to 63.9)	63.2 (62.0 to 64.4)	63.2 (62.0 to 64.4)	60.3 (59.1 to 61.6)	–3.4 (–6.0 to –0.7)	–2.1 (–3.9 to –0.3)	.02
South	66.5 (65.3 to 67.7)	64.9 (63.5 to 66.2)	65.1 (63.9 to 66.4)	63.1 (61.7 to 64.6)	–2.4 (–5.2 to 0.6)	–1.5 (–3.5 to 0.5)	.12
West	66.6 (64.8 to 68.4)	65.9 (64.3 to 67.5)	68.2 (66.6 to 69.7)	65.4 (62.9 to 67.8)	–4.0 (–8.3 to 0.4)	–2.8 (–5.7 to 0.1)	.07
Comorbidities, No.^d^							
None	65.6 (64.6 to 66.7)	65.1 (63.8 to 66.3)	64.7 (63.5 to 65.9)	64.1 (62.9 to 65.4)	–0.7 (–3.6 to 2.4)	–0.4 (–2.4 to 1.6)	.67
1-2	66.8 (65.6 to 68.0)	65.9 (64.5 to 67.2)	64.6 (63.1 to 66.0)	64.2 (62.7 to 65.5)	–1.4 (–4.7 to 2.1)	–0.9 (–3.1 to 1.3)	.43
≥2	66.4 (65.2 to 67.5)	65.6 (64.3 to 66.8)	67.1 (66.0 to 68.2)	63.0 (61.6 to 64.4)	–6.1 (–8.7 to –3.5)	–4.1 (–5.9 to –2.3)	<.001
Self-rated health							
Excellent or very good	66.8 (65.7 to 67.9)	65.3 (64.1 to 66.5)	65.7 (64.5 to 66.8)	63.6 (62.3 to 64.9)	–3.0 (–5.2 to –0.8)	–2.0 (–3.4 to –0.6)	.007
Good, fair, or poor	65.1 (64.2 to 66.0)	65.1 (64.2 to 66.0)	65.1 (64.2 to 65.9)	62.9 (61.8 to 64.0)	–2.6 (–5.3 to 0.2)	–1.7 (–3.5 to 0.1)	.06
Mental distress frequency, d^e^							
0 to <14	65.2 (64.4 to 66.0)	64.6 (63.7 to 65.4)	64.8 (64.0 to 65.6)	62.7 (61.7 to 63.6)	–2.5 (–4.5 to –0.4)	–1.6 (–3.0 to –0.2)	.02
≥14	67.3 (65.9 to 68.8)	67.1 (65.6 to 68.5)	66.8 (65.4 to 68.1)	65.1 (63.4 to 66.7)	–2.7 (–5.9 to 0.6)	–1.8 (–4.0 to 0.4)	.11
Physical distress frequency, d^f^							
0 to <14	66.7 (65.2 to 68.2)	67.5 (66.0 to 69.0)	67.8 (66.3 to 69.2)	64.4 (62.3 to 66.3)	–2.0 (–4.0 to 0.0)	–1.3 (–2.7 to 0.1)	.05
≥14	65.7 (65.0 to 66.4)	65.2 (64.5 to 65.9)	65.4 (64.7 to 66.1)	63.3 (62.4 to 64.1)	–4.9 (–8.5 to –1.1)	–3.3 (–5.8 to –0.8)	.01
Activity limitations frequency, d^g^							
0 to <14	68.2 (67.3 to 69.2)	67.5 (66.5 to 68.5)	67.4 (66.4 to 68.3)	65.7 (64.5 to 66.9)	–2.4 (–4.8 to 0.0)	–1.6 (–3.2 to –0.0)	.05
≥14	67.7 (66 to 69.4)	68.8 (67 to 70.5)	69.2 (67.6 to 70.7)	65.0 (62.9 to 67.0)	–5.4 (–9.3 to –1.5)	–3.8 (–6.5 to –1.1)	.007

^a^ Estimated as weighted percentage, where the numerator (current smokers [ie, those who smoked ≥100 cigarettes in their lifetime and now smoke every day or somedays] who reported that they stopped smoking for >1 day during the past 12 months because they were trying to quit smoking and former smokers [ie, ever smokers who currently smoked not at all] who quit during the past year [ie, last smoked a cigarette, “even 1 or 2 puffs,” within the past year]) was divided by the denominator (current smokers and former smokers who quit within the past year) and multiplied by 100.

^b^ Estimated as the ratio of 2020 vs 2019 predicted marginal probabilities minus 1, multiplied by 100, from a logistic regression model estimating the probability of a past-year quit attempt between 2011 and 2020, adjusted for age, sex, race and ethnicity, education level, marital status, region, number of comorbidities, smokeless tobacco use, heavy alcohol drinking, past 30-day mental distress frequency, and body mass index category (calculated as weight in kilograms divided by height in meters squared).

^c^ Estimated as the difference of 2020 vs 2019 marginal probabilities multiplied by 100, from a logistic regression model estimating the probability of a past-year quit attempt between 2011 and 2020, adjusted for age, sex, race and ethnicity, education level, marital status, region, number of comorbidities, smokeless tobacco use, heavy alcohol drinking, and body mass index (calculated as weight in kilograms divided by height in meters squared) category.

^d^ Self-reported history of heart attack (or myocardial infarction), angina or coronary heart disease, stroke, asthma, chronic obstructive pulmonary disease (emphysema or chronic bronchitis), arthritis, depressive disorder (including depression, major depression, dysthymia, or minor depression), kidney disease, diabetes, or cancer.

^e^ Self-reported mental distress frequency was based on responses to the following question: “Now thinking about your mental health, which includes stress, depression, and problems with emotions, for how many days during the past 30 days was your mental health not good?”

^f^ Self-reported physical distress frequency was based on responses to the following question: “Now thinking about your physical health, which includes physical illness and injury, for how many days during the past 30 days was your physical health not good?”

^g^ Among individuals who reported 1 or more days of past 30-day physical or mental distress, self-reported activity limitations frequency was based on responses to the following question: “During the past 30 days, for about how many days did poor physical or mental health keep you from doing your usual activities, such as self-care, work, or recreation?”

Recent successful cessation remained unchanged, at 5.9% (95% CI, 5.6%-6.3%) in 2019 and 6.3% (95% CI, 5.9%-6.7%) in 2020 (*P* = .36) but increased linearly over the entire study period, from 5.3% (95% CI, 5.0%-5.6%) in 2011 to 2020 (*P* < .001) (eTable 2 in the Supplement). Successful cessation levels did not change between 2019 to 2020 for any subgroup (eTable 3 in the Supplement).

### Changes in NRT Sales

**Figure 2.**
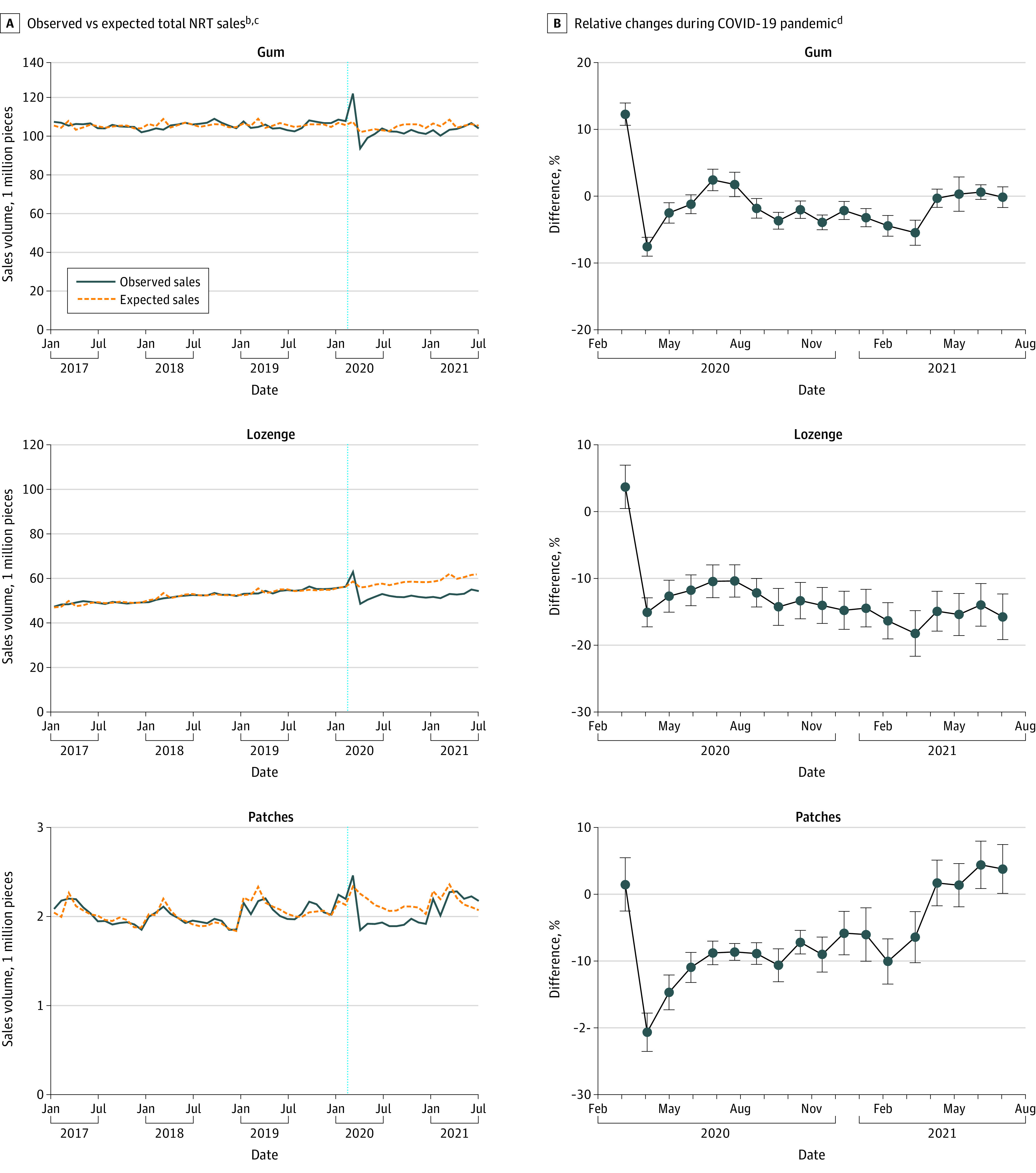
Nicotine Replacement Therapy (NRT) Sales Before and During the COVID-19 Pandemic^a^ ^a^The sample included 31 states (Alabama, Arizona, Connecticut, Florida, Georgia, Indiana, Kansas, Kentucky, Louisiana, Maryland, Michigan, Mississippi, Missouri, Nevada, New Jersey, North Carolina, Ohio, Oregon, Pennsylvania, South Carolina, Tennessee, Texas, Virginia, Washington, Wisconsin, California, Colorado, Illinois, Minnesota, New York, and Massachusetts). Two states (Arkansas and Oklahoma) with incomplete NRT data were excluded from the original sample. ^b^Expected trends during the COVID-19 pandemic (March 2020 to July 2021) were estimated based on trends estimated from the prepandemic period (January 2017 to February 2020) of observed NRT sales in 31 states from an interrupted time series regression model, adjusting for state and month fixed effects, inflation-adjusted NRT and cigarette prices, and state sex (men and women), marital status, age, race and ethnicity, education level, and household income composition. ^c^Total sales were observed and expected sales aggregated across 31 states in the sample. ^d^Relative change (%) was the mean difference in observed sales vs expected sales as a percentage change from expected sales. Error bars indicate 95% CIs.

In the prepandemic period, the observed mean (SD) NRT sales volume per sales period was 105.6 (66.2) million gum pieces, 51.9 (31.6) million lozenges, and 2.0 (1.1) million patches (eTable 4 in the Supplement). Compared with expected sales, observed sales during the COVID-19 pandemic were lower by 13.0% (95% CI, –13.7% to –12.3%) for lozenges, 6.4% (95% CI, –7.3% to –5.5%) for patches, and 1.2% (95% CI, –1.7% to –0.7%) for gum (eTable 5 in the Supplement). Of 18 four-week sales periods during the COVID-19 pandemic, a relative sales deficit persisted the longest for lozenges, at 17 periods (April 2020 to July 2021), followed by 13 periods for patches (April 2020 to March 2021) and 10 periods for gum (April to May 2020 and September 2020 to March 2021) (Figure 2).

## Discussion

This cross-sectional study found that, starting in 2020 quarter 2, the prevalence of past-year quit attempts among US adults decreased to its lowest level since 2011. Simultaneously, observed NRT retail sales across 31 US states decreased by a mean of 1% to 13% vs expected sales. These findings suggest an immediate decrease in serious quitting activity among US smokers after the COVID-19 pandemic onset, a decrease that persisted throughout 2020. NRT sales results suggest a continuation of this trend in 2021 quarter 1 but potentially a return to expected levels in 2021 quarter 2 based on NRT gum and patch sales. These findings are consistent with a report^12^ of a sustained decrease in cessation assistance calls to state quit lines from 2020 quarter 2 to 2021 quarter 1, followed by an increase in 2021 quarter 2.

Decreases in prevalence of quit attempts were steepest among many groups known to have experienced the most severe economic and health effects of the pandemic: Black individuals, those with 2 or more comorbidities and past 30-day frequent physical distress and activity limitations, those with lower education levels, middle-aged individuals, and women.^13,14,15^ Quit attempts may have decreased with increased COVID-19–related stress coping^6^ or health care disruptions that reduced clinical access to cessation treatments.^16^ Decreases in quit attempts may be associated with future successful cessation rates given that a typical smoker attempts to quit a mean of 6 times before achieving sustained abstinence.^1,17^

### Limitations

This study has several limitations. Past-year quit attempt prevalence in 2020 may partly reflect attempts in 2019, before the COVID-19 pandemic. Although BRFSS estimates are weighted for nonresponse and self-reported cessation is generally accurate, residual survey biases may remain.^10,18^ However, BRFSS maintained a steady response rate vs past years, and there was no indication of differential nonresponse rates in 2020 vs 2019 that could have biased quit attempt prevalence downward (eg, individuals with lower education levels and relatively lower quit attempt prevalence were not more likely to respond, and Black individuals with relatively higher quit attempt prevalence rates were not less likely to respond). Some states paused interviews during shutdowns, and survey interview distribution by quarter varied in 2020 vs past years. However, regression analyses were adjusted for interview quarter, and results were similar compared with results that did not include adjustment for quarter. Additionally, the survey’s telephone-based administration mode remained unchanged during the pandemic, unlike other national surveys.^10,19^

NRT sales data have limitations given that those sales serve as a proxy for consumption but do not equate to it, and sales data did not include all states, online sales, or sales in health care settings. Additionally, while there is no evidence that NRT sales trends during the COVID-19 pandemic may have differed from non-NRT smoking-cessation treatments (eg, prescription pharmacotherapies and behavioral counseling), significant increases have been reported in substance abuse and mental health–related visits via telehealth and prescription drug expenditures via mail orders and home health care during 2020.^20,21^ It is unknown to what extent this general shift to alternative treatment models applied to non-NRT cessation treatments or whether this trend may have offset the decrease in NRT sales shown in our study. Additionally, sales trends of individual NRT products were not completely independent of one another because combination NRT use is a clinically recommended smoking cessation treatment.^17^ Despite these limitations, the Nielsen sales data used in this study were collected in a consistent manner before and during the COVID-19 pandemic from a representative sample of retailers in nearly two-thirds of US states.

## Conclusions

Findings from 2 independent data sources in this cross-sectional study suggest that serious cessation activity decreased immediately after the COVID-19 pandemic onset and remained depressed through 2021 quarter 1. These results, when taken together with reports of increased cigarette sales during the pandemic,^22^ suggest the urgent need to reengage smokers in evidence-based quitting strategies, especially among individuals experiencing disproportionately negative outcomes during the pandemic.

## Notes

Supplement. **eTable 1.** Sample Characteristics of Current Smokers and Former Smokers Who Quit in Last Year **eTable 2.** Prevalence of Recent Successful Smoking Cessation, 2011-2020 **eTable 3.** Prevalence of Recent Successful Smoking Cessation, 2017-2020 **eTable 4.** 4-Week Nicotine Replacement Therapy Retail Sales Data and State Sociodemographic Composition in 31 US States Before and During the COVID-19 Pandemic **eTable 5.** Changes in 4-week Nicotine Replacement Therapy Retail Sales in 31 US States Before and During the COVID-19 Pandemic
