# Supplementary material for: Changes in Smoking Cessation–Related Behaviors Among US Adults During the COVID-19 Pandemic
Source: JAMA Netw Open. 2022 Aug 1;5(8):e2225149. doi: 10.1001/jamanetworkopen.2022.25149 (PMC9344362; doi:10.1001/jamanetworkopen.2022.25149)
Supplement: Supplement. — eTable 1. Sample Characteristics of Current Smokers and Former Smokers Who Quit in Last Year eTable 2. Prevalence of Recent Successful Smoking Cessation, 2011-2020 eTable 3. Prevalence of Recent Successful Smoking Cessation, 2017-2020 eTable 4. 4-Week Nicotine Replacement Therapy Retail Sales Data and State Sociodemographic Composition in 31 US States Before and During the COVID-19 Pandemic eTable 5. Changes in 4-week Nicotine Replacement Therapy Retail Sales in 31 US States Before and During the COVID-19 Pandemic [file jamanetwopen-e2225149-s001.pdf]

Supplemental Online Content

Bandi P, Asare S, Majmundar A, et al. Changes in smoking cessation–related behaviors among US adults during the COVID-19 pandemic. *JAMA Netw Open*. 2022;5(8):e2225149. doi:10.1001/jamanetworkopen.2022.25149

eTable 1. Sample Characteristics of Current Smokers and Former Smokers Who Quit in Last Year

eTable 2. Prevalence of Recent Successful Smoking Cessation, 2011-2020

eTable 3. Prevalence of Recent Successful Smoking Cessation, 2017-2020

eTable 4. 4-Week Nicotine Replacement Therapy Retail Sales Data and State Sociodemographic Composition in 31 US States Before and During the COVID-19 Pandemic

eTable 5. Changes in 4-week Nicotine Replacement Therapy Retail Sales in 31 US States Before and During the COVID-19 Pandemic

This supplemental material has been provided by the authors to give readers additional information about their work.

© 2022 Bandi P et al. *JAMA Network Open.*

eTable 1. Sample Characteristics of Current Smokers and Former Smokers Who Quit in Last Year

	No (weighted %)			
**Characteristic**	**2011-2020 (n=788,008)**	**2019 (n=67,425)**	**2020 (n=62,480)**	***2019 vs. 2020 change P* value** [a]
Quarter				
Q1	178380 (23.0)	12217 (18.9)	14568 (22.6)	<0.001
Q2	197720 (25.7)	18576 (29.8)	15757 (27.0)	
Q3	198716 (25.6)	17049 (26.0)	14021 (23.1)	
Q4	196678 (25.8)	16647 (25.3)	16406 (27.3)	
Age-group (years)				
18-24	48511 (12.0)	3654 (9.5)	3206 (8.8)	0.04
25-44	243061 (42.5)	21562 (43.5)	21046 (43.0)	
45-64	346915 (35.5)	27764 (35.0)	25417 (35.4)	
65+	149521 (10.0)	14445 (12.0)	12811 (12.8)	
Sex				
Female	413271 (44.3)	33560 (44.3)	30871 (43.8)	0.32
Male	374519 (55.7)	33865 (55.7)	31609 (56.2)	
Race/ethnicity				
White persons	583306 (68.9)	49096 (67.4)	45137 (67.0)	0.37
Black persons	69638 (12.7)	5521 (12.8)	5080 (13.0)	
Hispanic persons	56829 (14.1)	5333 (15.1)	5047 (15.2)	
AI/AN persons	23763 (1.8)	2054 (2.0)	2066 (1.7)	
Asian persons	9614 (2.5)	914 (2.8)	913 (3.1)	
Education level				
<HS or HS	395862 (55.9)	32887 (54.7)	30729 (55.0)	0.85
Some college	242891 (31.6)	21428 (32.2)	19766 (31.9)	
College	147516 (12.6)	12922 (13.0)	11822 (13.1)	
No. of comorbidities [b]				
None	267516 (40.0)	21257 (36.9)	20970 (38.1)	0.21
1-2 conditions	207917 (26.7)	17932 (27.6)	16825 (26.9)	
>=2	278181 (29.2)	24927 (31.2)	22064 (30.8)	
Missing	34394 (4.2)	3309 (4.4)	2621 (4.3)	
Heavy alcohol drinking [c]				
Yes	85673 (11.8)	7925 (12.7)	7667 (12.8)	0.55
No	667704 (82.8)	56758 (82.4)	51988 (82.0)	
Missing	34631 (5.4)	2742 (4.9)	2825 (5.2)	

© 2022 Bandi P et al. *JAMA Network Open.*

Marital status				
Married	296590 (37.1)	23761 (36.4)	22579 (37.1)	0.24
Not married	487457 (62.9)	43247 (63.6)	39482 (62.9)	
Region				
Northeast	139269 (16.5)	9973 (13.9)	10527 (15.4)	<0.001
Midwest	219762 (23.8)	19574 (24.2)	18843 (23.7)	
South	258777 (40.5)	22813 (41.7)	19225 (41.9)	
West	160243 (19.3)	13974 (20.3)	12983 (19.1)	
Self-rated health				
Excellent or very good	290945 (39.1)	22977 (36.0)	25599 (43.6)	<0.001
Good, fair, or poor	494356 (60.9)	44244 (64.0)	36682 (56.4)	
Mental distress frequency [d]				
0-<14 days	606744 (77.8)	49955 (75.0)	47196 (76.7)	<0.001
≥14 days	165666 (22.2)	15827 (25.1)	13904 (23.3)	
Physical distress frequency [e]				
0-<14 days	609381 (81.8)	51010 (80.6)	50203 (84.1)	<0.001
≥14 days	161612 (18.2)	14655 (19.4)	10831 (15.9)	
Activity limitations frequency [f]				
0-<14 days	360620 (76.8)	32203 (75.5)	27770 (75.6)	0.90
≥14 days	125172 (23.2)	11637 (24.5)	9436 (24.4)	
Weight status (BMI, kg/m2)				
Normal weight (≤18.0 kg/m2)	286491 (35.8)	23578 (33.7)	21162 (33.3)	<0.001
Overweight (25.0-29.9 kg/m2)	256110 (32.3)	21464 (32.0)	19939 (30.9)	
Obese (≥30 kg/m2)	212302 (27.3)	19548 (29.8)	18136 (30.0)	
Missing	33105 (4.5)	2835 (4.6)	3243 (5.8)	
Smokeless tobacco use status				
Current	48863 (7.0)	4722 (7.6)	4047 (6.7)	0.003
noncurrent	736887 (93.0)	62477 (92.4)	58206 (93.3)	

Abbreviation: HS: High school; Q: Quarter

[a] Chi-squared test was used to assess difference between 2019 and 2020 in sample characteristics.

[b] Self-reported history of heart attack (aka myocardial infarction), angina or coronary heart disease, stroke, asthma, chronic obstructive pulmonary disease (COPD, emphysema or chronic bronchitis), arthritis, depressive disorder (including depression, major depression, dysthymia, or minor depression), kidney disease, diabetes, or cancer.
[c] Self-reported adult men having more than 14 drinks per week and adult women having more than 7 drinks per week in past 30-days.

© 2022 Bandi P et al. *JAMA Network Open.*

[d] Self-reported mental distress frequency based on response to question, "Now thinking about your mental health, which includes stress, depression, and problems with emotions, for how many days during the past 30 days was your mental health not good?"
[e] Self-reported physical distress frequency based on response to question, "Now thinking about your physical health, which includes physical illness and injury, for how many days during the past 30 days was your physical health not good?
[f] Among those who reported ≥1 day of past 30-days physical or mental distress, self-reported activity limitations frequency based on response to question, "During the past 30 days, for about how many days did poor physical or mental health keep you from doing your usual activities, such as self-care, work, or recreation?

© 2022 Bandi P et al. *JAMA Network Open.*

eTable 2. Prevalence of Recent Successful Smoking Cessation, 2011-2020

	**Recent successful cessation** [a]	**Annual Absolute change** [b]	
**Year**	**% (95% CI)**	**% points (95% CI)**	***P* value**
2011	5.3 (5.0, 5.6)	--	--
2012	5.1 (4.8, 5.4)	-0.3 (-0.7, 0.1)	0.21
2013	5.7 (5.4, 6.0)	0.6 (0.2, 1.0)	0.01
2014	5.6 (5.3, 5.9)	-0.1 (-0.5, 0.3)	0.64
2015	5.7 (5.4, 6.0)	0.2 (-0.2, 0.5)	0.51
2016	5.7 (5.4, 6.1)	-0.1 (-0.4, 0.3)	0.84
2017	5.3 (5.0, 5.7)	-0.4 (-1.0, 0.2)	0.12
2018	5.8 (5.5, 6.2)	0.5 (-0.1, 1.1)	0.06
2019	5.9 (5.6, 6.3)	0.2 (-0.4, 0.7)	0.58
2020	6.3 (5.9, 6.7)	0.3 (-0.3, 0.9)	0.36
2011 vs. 2020 Absolute change, % points (95% CI)		0.8 (0.2, 1.4)	0.002

[a] Estimated as percentage where (numerator = former smokers who reported last smoking a cigarette, even one or two puffs, > 6 months but less than 1 year ago / denominator = current smokers [i.e. smoked 100+ cigarettes in lifetime, now smoke every day or somedays] and former smokers [i.e. ever smokers who now smoke "not at all"] who quit during the past year [i.e. last smoked a cigarette, even one or two puffs within the past year]) x 100.

[b] Estimated as (Difference of current vs. prior year predicted marginal probabilities) x 100 from logistic regression model predicting probability of recent successful cessation between 2011-2020, adjusted for age, sex, race/ethnicity, education level, marital status, region, number of comorbidities, smokeless tobacco use, heavy alcohol drinking,

© 2022 Bandi P et al. *JAMA Network Open.*

eTable 3. Prevalence of Recent Successful Smoking Cessation, 2017-2020

	Recent Successful Cessation,[a] % (95% CI)				2019-2020	
	**2017 (n=75358)**	**2018 (n=73159)**	**2019 (n=67425)**	**2020 (n=62480)**	**Absolute difference 2020 vs 2019[b]**	***P* value**
	**% (95 % CI)**	**% (95 % CI)**	**% (95 % CI)**	**% (95 % CI)**	**% points (95% CI)**	
Total	5.3 (5, 5.7)	5.8 (5.5, 6.2)	5.9 (5.6, 6.3)	6.3 (5.9, 6.7)	0.3 (-0.3, 0.9)	0.36
Quarter						
Q1	5.6 (4.9, 6.3)	5.3 (4.6, 6.0)	5.8 (5.1, 6.7)	5.3 (4.7, 6.0)	-0.6 (-1.6, 0.4)	0.30
Q2	5.5 (4.9, 6.1)	5.8 (5.1, 6.6)	5.8 (5.2, 6.5)	6.2 (5.5, 7.0)	0.3 (-0.7, 1.3)	0.57
Q3	4.9 (4.3, 5.5)	6.3 (5.6, 7.2)	6.6 (6.0, 7.4)	7.0 (6.0, 8.0)	0.6 (-0.6, 1.8)	0.34
Q4	5.4 (4.7, 6.3)	5.9 (5.3, 6.6)	5.4 (4.8, 6.1)	6.4 (5.6, 7.3)	0.8 (-0.4, 1.9)	0.20
Age-group (years)						
18-24	5.9 (4.9, 7.2)	7.1 (5.9, 8.5)	8.0 (6.8, 9.4)	9.5 (7.9, 11.4)	0.7 (-1.4, 2.9)	0.49
25-44	5.9 (5.4, 6.5)	6.6 (6.1, 7.2)	6.7 (6.2, 7.4)	7.7 (7.0, 8.5)	1.0 (0.0, 2.0)	0.06
45-64	4.6 (4.1, 5.0)	4.7 (4.2, 5.2)	4.6 (4.2, 5.1)	4.3 (3.8, 4.9)	-0.4 (-1.1, 0.4)	0.37
65+	4.9 (4.1, 6.0)	5.0 (4.2, 6.0)	5.1 (4.2, 6.1)	4.7 (4.1, 5.5)	-0.6 (-1.7, 0.6)	0.38
Sex						
Female	5.6 (5.1, 6.1)	5.4 (5.0, 6.0)	5.4 (4.9, 5.9)	5.7 (5.1, 6.2)	0.1 (-0.7, 0.9)	0.74
Male	5.1 (4.7, 5.6)	6.1 (5.6, 6.6)	6.3 (5.8, 6.8)	6.7 (6.2, 7.4)	0.4 (-0.4, 1.2)	0.36
Race/ethnicity						
White persons	5.6 (5.3, 6.0)	5.8 (5.4, 6.1)	6.2 (5.8, 6.6)	6.5 (6.1, 7.0)	0.4 (-0.2, 1.0)	0.19
Black persons	3.8 (2.9, 5.1)	4.0 (3.3, 4.9)	4.3 (3.4, 5.3)	3.4 (2.6, 4.5)	-1.0 (-2.3, 0.4)	0.16
Hispanic persons	5.4 (4.5, 6.5)	7.2 (5.9, 8.7)	6.0 (5.1, 7.2)	7.6 (6.2, 9.4)	1.3 (-0.7, 3.3)	0.22
AI/AN persons	3.3 (2.4, 4.6)	4.9 (3.3, 7.1)	5.1 (3.6, 7.3)	4.4 (3.1, 6.4)	-0.7 (-3.2, 1.9)	0.61
Asian persons	6.2 (4.0, 9.4)	9.2 (5.8, 14.3)	8.7 (5.5, 13.6)	8.3 (5.4, 12.5)	-1.7 (-6.6, 3.2)	0.50
Education level						
<HS or HS	4.2 (3.8, 4.6)	4.5 (4.1, 5.0)	4.8 (4.4, 5.3)	5.0 (4.5, 5.6)	0.2 (-0.6, 1.0)	0.59
Some college	5.9 (5.3, 6.6)	6.3 (5.7, 7.0)	6.0 (5.5, 6.7)	6.9 (6.1, 7.7)	0.8 (-0.2, 1.8)	0.13
College	9.0 (8.0, 10.0)	9.8 (8.9, 10.9)	10.0 (9.0, 11.1)	10.0 (9, 11.1)	-0.5 (-2.1, 1.0)	0.49
Region						

© 2022 Bandi P et al. *JAMA Network Open.*

Northeast	5.4 (4.7, 6.2)	6.2 (5.3, 7.1)	5.9 (5.2, 6.8)	6.4 (5.7, 7.3)	0.5 (-0.7, 1.6)	0.43
Midwest	5.0 (4.5, 5.5)	4.5 (4.1, 5.0)	5.6 (5.1, 6.2)	5.7 (5.1, 6.3)	-0.3 (-1.0, 0.5)	0.54
South	5.0 (4.5, 5.6)	5.5 (5.0, 6.1)	5.3 (4.8, 5.9)	5.9 (5.3, 6.6)	0.6 (-0.4, 1.6)	0.24
West	6.3 (5.5, 7.2)	7.6 (6.7, 8.5)	7.4 (6.6, 8.4)	7.8 (6.6, 9.1)	0.2 (-1.4, 1.8)	0.80
No. of comorbidities [c]						
None	5.7 (5.2, 6.3)	6.5 (5.9, 7.2)	7.0 (6.4, 7.7)	7.2 (6.5, 8.0)	0.0 (-1.0, 1.0)	1.0
1-2 conditions	5.9 (5.3, 6.5)	5.9 (5.3, 6.5)	5.8 (5.2, 6.5)	6.4 (5.7, 7.3)	0.5 (-0.7, 1.6)	0.42
≥2	4.6 (4.1, 5.2)	4.8 (4.3, 5.3)	4.8 (4.4, 5.4)	5.1 (4.5, 5.7)	0.3 (-0.5, 1.1)	0.50

HS: 95% CI: 95% confidence intervals; High school; Q: Quarter

[a] Estimated as percentage where (numerator = former smokers who reported last smoking a cigarette, even one or two puffs, > 6 months but less than 1 year ago / denominator = current smokers [i.e. smoked 100+ cigarettes in lifetime, now smoke every day or somedays] and former smokers [i.e. ever smokers who now smoke "not at all"] who quit during the past year [i.e. last smoked a cigarette, even one or two puffs within the past year]) x 100.

[b] Estimated as (Difference of 2020 vs. 2019 adjusted predicted marginal probabilities) x 100 from logistic regression model predicting probability of recent successful cessation between 2011-2020, adjusted for age, sex, race/ethnicity, education level, marital status, region, number of comorbidities, smokeless tobacco use, heavy alcohol drinking, past 30-day poor mental health days, and weight status. Only absolute changes are shown as relative changes are inflated due to low absolute levels of recent successful cessation.

[c] Self-reported history of heart attack (aka myocardial infarction), angina or coronary heart disease, stroke, asthma, chronic obstructive pulmonary disease (COPD, emphysema or chronic bronchitis), arthritis, depressive disorder (including depression, major depression, dysthymia, or minor depression), kidney disease, diabetes, or cancer.

© 2022 Bandi P et al. *JAMA Network Open.*

eTable 4. 4-Week Nicotine Replacement Therapy Retail Sales Data and State Sociodemographic Composition in 31 US States Before and During the COVID-19 Pandemic[a]

Characteristic	Before the COVID-19 pandemic (January 2017-February 2020) (n=1271) Mean (SD)	During the COVID-19 pandemic (March 2020-July 2021) (n=558) Mean (SD)	Difference	*P* value
**Retail sales data**				
NRT sales volume (million pieces)				
Gum	105.6	103.2	-2.3	.47
	(66.2)	(62.4)	(3.2)	
Lozenge	51.9	52.7	0.7	.64
	(31.6)	(31.5)	(1.6)	
Patches	2.03	2.05	0.02	.68
	(1.14)	(1.16)	(0.06)	
Prices of NRTs, Feb. 2020 $ per piece				
Gum	0.32	0.31	-0.02	< .001
	(0.02)	(0.02)	(0.00)	
Lozenge	0.38	0.36	-0.02	< .001
	(0.02)	(0.02)	(0.00)	
Patches	2.02	1.95	-0.07	< .001
	(0.06)	(0.05)	(0.00)	
Price of cigarettes, Feb. 2020 $ per pack	6.95	7.29	0.34	< .001
	(1.64)	(1.68)	(0.08)	
**State composition**				
Male	0.49	0.49	0.00	.61
	(0.01)	(0.01)	(0.00)	
Married	0.52	0.52	0.00	.36
	(0.02)	(0.03)	(0.00)	
Age distribution				
<25 y	0.30	0.29	-0.01	< .001
	(0.02)	(0.02)	(0.00)	
25-44 y	0.25	0.25	-0.00	0.008
	(0.02)	(0.02)	(0.00)	
45-64 y	0.27	0.26	-0.01	< .001
	(0.02)	(0.02)	(0.00)	
>64 y	0.18	0.20	0.02	< .001
	(0.02)	(0.02)	(0.00)	
Racial distribution				
White persons	0.80	0.79	-0.00	.31
	(0.08)	(0.08)	(0.00)	
Black persons	0.13	0.13	0.00	.93
	(0.09)	(0.09)	(0.00)	
Asian persons	0.05	0.05	0.00	.14
	(0.03)	(0.04)	(0.00)	
Other	0.03	0.03	0.00	.34
	(0.02)	(0.02)	(0.00)	
Education distribution				

© 2022 Bandi P et al. *JAMA Network Open.*

High school diploma	0.28	0.28	-0.00	.17
	(0.04)	(0.04)	(0.00)	
Some college	0.27	0.26	-0.01	< .001
	(0.03)	(0.03)	(0.00)	
College or higher degree	0.31	0.33	0.02	< .001
	(0.06)	(0.06)	(0.00)	
Income distribution				
<$10 000	0.18	0.21	0.03	< .001
	(0.03)	(0.04)	(0.00)	
$10 000-$29 999	0.14	0.12	-0.02	< .001
	(0.04)	(0.03)	(0.00)	
$30 000-$59 999	0.22	0.20	-0.02	< .001
	(0.03)	(0.03)	(0.00)	
$60 000-$149 999	0.33	0.33	-0.00*	.03
	(0.04)	(0.04)	(0.00)	
$150 000+	0.12	0.13	0.02	< .001
	(0.05)	(0.05)	(0.00)	

Abbreviation: SD: Standard Deviation

[a] 31 states in sample (Alabama, Arizona, Connecticut, Florida, Georgia, Indiana, Kansas, Kentucky, Louisiana, Maryland, Michigan, Mississippi, Missouri, Nevada, New Jersey, North Carolina, Ohio, Oregon, Pennsylvania, South Carolina, Tennessee, Texas, Virginia, Washington, Wisconsin, California, Colorado, Illinois, Minnesota, New York, and Massachusetts). Two states (Arkansas and Oklahoma) with incomplete NRT data were dropped from original sample.

© 2022 Bandi P et al. *JAMA Network Open.*

eTable 5. Changes in Nicotine Replacement Therapy Retail Sales in 31 US States Before and During the COVID-19 Pandemic[a]

	**Gum**	**Lozenge**	**Patches**
**NRT sales changes before vs. during COVID-19 pandemic** [b]	**(n=1829)**	**(n=1829)**	**(n=1829)**
	Millions of Pieces (95% CI)	Millions of Pieces (95% CI)	Millions of Pieces (95% CI)
Trend before COVID-19 pandemic, (Jan 2017 – February 2021)	0.05 (0.01, 0.09)	0.22 (0.21, 0.24)	0.01 (0.01, 0.01)
Level change at COVID-19 onset (March 2020)	6.66 (-4.14, 17.45)	9.35 (3.74, 14.95)	-0.97 (-1.29, -0.65)
Trend change during COVID-19 pandemic (March 2020 – July 2021)	-0.17 (-0.38, 0.05)	-0.31 (-0.42, -0.19)	0.02 (0.01, 0.02)
**Observed vs. expected** [c] **NRT sales during COVID-19 pandemic (March-July 2021)**	**(n=558)**	**(n=558)**	**(n=558)**
Absolute Change	Millions of Pieces (95% CI)	Millions of Pieces (95% CI)	Millions of Pieces (95% CI)
	-1.8 (-2.46, -1.14)	-6.07 (-6.42, -5.73)	-0.11 (-0.13, -0.09)
Relative Change [d]	% (95% CI)	% (95% CI)	% (95% CI)
	-1.20 (-1.7, -0.7)	-13.0 (-13.7, -12.3)	-6.4 (-7.3, -5.5)

[a] 31 states in sample (Alabama, Arizona, Connecticut, Florida, Georgia, Indiana, Kansas, Kentucky, Louisiana, Maryland, Michigan, Mississippi, Missouri, Nevada, New Jersey, North Carolina, Ohio, Oregon, Pennsylvania, South Carolina, Tennessee, Texas, Virginia, Washington, Wisconsin, California, Colorado, Illinois, Minnesota, New York, and Massachusetts). Two states (Arkansas and Oklahoma) with incomplete NRT data were dropped from original sample.

[b] Regression coefficients scaled up to reflect total across 31 states were estimated from interrupted time series regression model of observed state-level aggregated 4-week NRT sales volume (million pieces), adjusting for state- and month- fixed effects, inflation-adjusted NRT and cigarette prices, and state's sex (male, female), marital status, age, race, education, and household income composition.

[c] Expected trends during COVID-19 (March 2020-July 2021) were predicted based on trends estimated from pre-pandemic period (January 2017–February 2020).

[d] Relative change (%) was mean difference in observed sales vs. expected sales as a percentage change from expected sales.

© 2022 Bandi P et al. *JAMA Network Open.*
